# Embelin: A multifaceted anticancer agent with translational potential in targeting tumor progression and metastasis

**DOI:** 10.17179/excli2023-6590

**Published:** 2023-12-13

**Authors:** Adithya Jayaprakash Kamath, Alda Sara Chandy, Aina Ann Joseph, Jaggiah N. Gorantla, Asawari Dilip Donadkar, Lekshmi R. Nath, Javad Sharifi-Rad, Daniela Calina

**Affiliations:** 1 Department of Pharmacognosy, Amrita School of Pharmacy, Amrita Vishwa Vidyapeetham, AIMS Health Sciences Campus, Kochi-682 041, India; 2 Department of Pharmaceutics, Amrita School of Pharmacy, Amrita Vishwa Vidyapeetham, AIMS Health Sciences Campus, Kochi-682 041, India; 3 Department of Chemistry, Wayne State University, Detroit-48202, Michigan, USA; 4 Facultad de Medicina, Universidad del Azuay, Cuenca, Ecuador; 5 Department of Clinical Pharmacy, University of Medicine and Pharmacy of Craiova, 200349 Craiova, Romania

**Keywords:** Embelin, Embelia ribes, cancer, antitumor mechanisms, bioavailability, toxicity of Embelin

## Abstract

Embelin, a natural para-benzoquinone product, is derived from plants of the Embelia genus, particularly *Embelia ribes* Burm.f. A staple in traditional medicinal formulations for centuries, Embelin's pharmacological actions are attributed to the hydroxyl benzoquinone present in its structure. Its therapeutic potential is bolstered by unique physical and chemical properties. Recently, Embelin, recognized as a non-peptidic, cell-permeable small inhibitor of the X-linked inhibitor of apoptosis protein (XIAP), has garnered significant attention for its anticancer activity. It demonstrates various anticancer mechanisms, such as apoptosis induction, cell cycle arrest, and autophagy, in different cancer types. Additionally, Embelin modulates several signal transduction pathways, including NF-κB, PI3Kinase/AKT, and STAT3, effectively inhibiting the proliferation of diverse cancer cell lines. This literature review illuminates the anticancer potential of Embelin, detailing its mechanisms of action and prospective clinical applications, based on relevant scientific literature from the past decade sourced from various electronic databases.

See also the Graphical abstract(Fig. 1).

## Introduction

Cancer is a well-recognized global health concern, lacking a sustainable resolution to date (EU, 2023[20]). According to the latest GLOBOCAN reports, cancer mortality will increase 1.6 times by 2040 (Sung et al., 2021[72]). The statistics significantly show that it is essential to develop a permanent global solution for this non-communicable disease (Huang et al., 2022[28]). As a part of making the world cancer-free, most countries have already started investing their efforts to attain this vision through various project schemes (EU, 2023[20]; Government of India, 2023[23]). Increasing knowledge of the diverse mechanisms of cancer has led the scientific community to explore different strategies for the prevention and treatment of cancer (Debela et al., 2021[14]). Existing anticancer drugs possess different limitations like side effects such as immunosuppression, cardiotoxicity, severe hair loss, mucositis, myelosuppression, systemic toxicity due to off-target deposition of drugs, increased resistance of cancer cells to the therapeutic agents (Singh et al., 2016[67]). Complementary therapy for cancer is gaining recognition as an emerging approach that offers potential as a sustainable solution for managing chronic conditions. Extensive research is being conducted on various phytomolecules to develop effective cancer therapies. These natural compounds hold promise for their potential anti-cancer properties and may provide alternative or adjunctive treatments to conventional cancer therapies. Exploring phytomolecules as potential therapies against cancer is an important area of study in improving cancer management (Rahman et al., 2021[61]). Embelin is a phytochemical currently being investigated for its potential anti-cancer properties in various malignancies. Embelin is a natural benzoquinone obtained from *Embelia ribes* Burm. mainly, is used widely in Ayurvedic, Siddha, and Unani medicinal formulations (Vijayan and Raghu, 2021[74]). Conventionally, it is used for its anthelmintic, astringent, stimulant, antipyretic, expectorant, hypoglycemic, antihyperlipidemic, antihypertensive, anti-diarrheal, antibiotic, contraceptive, painkiller, anti-erythrogenic, anticancer and antioxidant properties (Chitra et al., 2003). Moreover, the plant is also used to treat leprosy, hepatic ailments, loss of appetite, dropsy, haemorrhoids and leukoderma (Alam et al., 2015[2]). Many preclinical studies have demonstrated the diverse mechanisms by which Embelin may contribute to preventing different types of cancer. This review is dedicated to exploring the potential of Embelin as an anti-cancer agent, shedding light on its various mechanisms of action. Additionally, it provides a concise overview of the factors that influence the clinical translation of Embelin as a promising therapeutic option for cancer treatment. By examining the multiple anti-cancer mechanisms of Embelin and addressing the factors affecting its clinical application, this review aims to enhance our understanding of the potential of Embelin in the fight against cancer.

## Review Methodology

**Figure 2:**
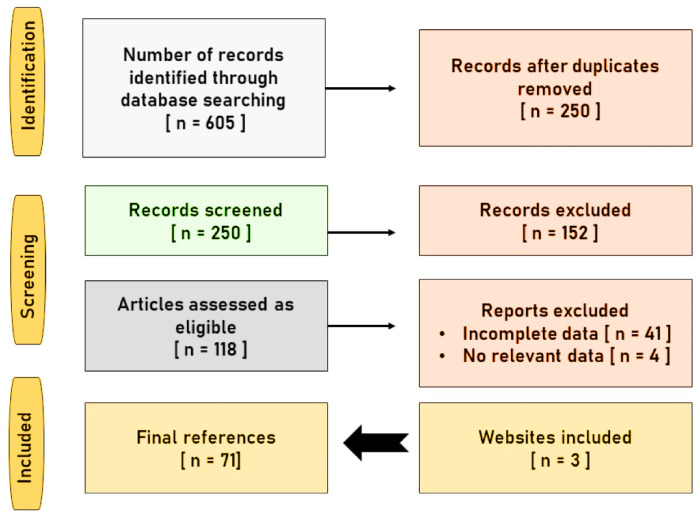
Illustrative diagram regarding the methodology adopted for the literature analysis

A thorough literature analysis was conducted using PubMed/MedLine, Google Scholar, Scopus, TRIP database and Science Direct. The diagram depicted in Figure 2(Fig. 2) describes the methodology followed for the present review. The literature review analysis was conducted per the directions provided by Page et al. (2021[53]). The keywords were searched separately using 'AND' as the Boolean operator. The keywords for the primary screening included 'Embelin', 'Anticancer mechanism', 'Vidanga', 'False black pepper', 'Cancer', 'Phytochemistry', 'Bioavailability', 'Pharmacokinetics', 'Toxicity' and 'Safety'. A total of 605 articles were identified in the preliminary screening. A total of 250 articles were identified after removing the duplicates. Among these, a total of 118 articles were found to be eligible. Among these, 41 articles were excluded as they contained inaccurate and incomplete data. A total of 4 abstracts were excluded as they didn't provide any relevant, novel and complete data. A total of 64 references (61 articles and 3 websites) were included in this article. 

## An Overview of the Ethnobotany of Embelin

Embelin (IUPAC name- 2,5-dihydroxy-3-undecyl-1,4-benzoquinone) is an active constituent of the plants under the *Embelia* genus (family: Myrsinaceae). On analyzing different species of *Embelia* genus namely-* Embelia ribes, Embelia tsjeriam-cottam, Embelia basal, Embelia adnata,* and *Embelia gardneriana *by High-Performance Thin Layer Chromatography (HPTLC) technique, it was found that *E. ribes *has the highest concentration of Embelin compared to other species (Kamble et al., 2020[35]). The plant *E. ribes* is typically seen in forest areas (at an altitude of or above 400-1200 m) semi-evergreen to evergreen in nature, including Sri Lanka, China, Malaysia and India. In India, the plant is predominantly seen in the Sahyadri Hills of Tamil Nadu, Karnataka and Kerala (Kundap et al., 2017[39]; Alam et al., 2015[2]). The erubescent-colored bulbous berries* of E. ribes* is popularly known as 'Vidanga', 'False Black Pepper' or 'Vai Vidang'. Embelin is the major constituent in all the parts of *E. ribe*. (Durg et al., 2017[16]). 

Kamble et al. (2020[35]) conducted a phytochemical screening of Vidanga collected from different regions of the Western Ghats of India. The methanolic extracts show higher phenolic and alkaloidal content, whereas the ethanolic extracts revealed higher flavonoid content. It was noticed that all the higher amounts of phenols, alkaloids and flavonoids were extracted from the plants belonging to the Nagavalli village, Shimoga district, Karnataka (India) (Kamble et al., 2020[35]). Another study conducted at Hamdard University, New Delhi, India, revealed that the aqueous extract of the berries of *E. ribes *consists of essential oils, alkaloids, proteins, flavonoids, carbohydrates, phenolic components, and saponins (Sharma et al., 2022[65]). Structurally, Embelin consists of two carbonyl groups, a methine group, and two hydroxyl groups. The undecyl alkyl chain offers compound lipophilicity and cell permeability (Othman et al., 2020[52]).

## Phytochemistry of Embelin and its Derivatives

**Figure 3:**
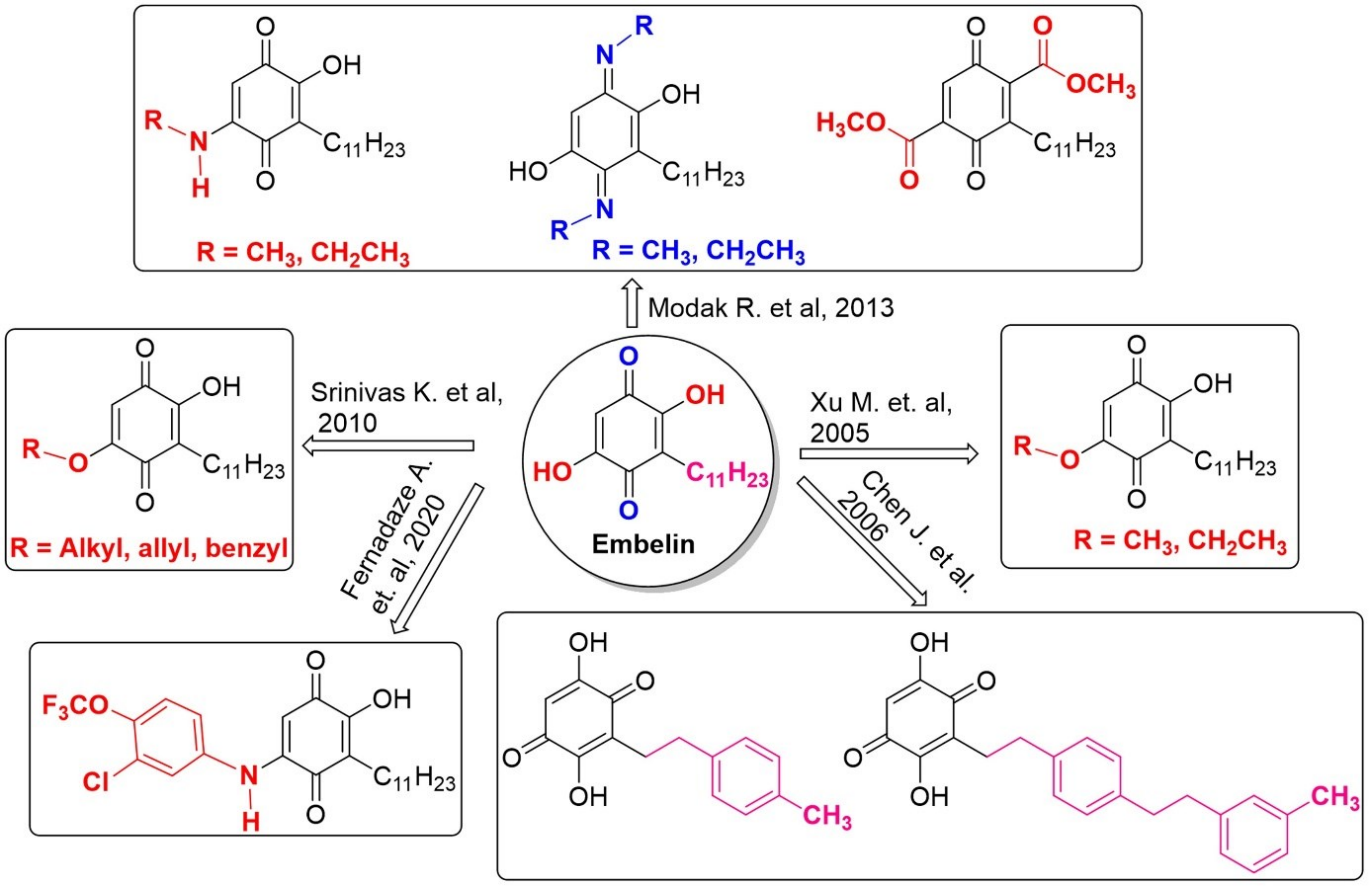
Structures of anticancer Embelin and its derivatives. Modification at carbonyl (blue), hydroxyl (red) and long-chain alkyl (pink)

Benzoquinone core moiety in the Embelin, is responsible for the anti-cancer activity. Molecular docking and structure-activity relationship studies show the carbonyl, hydroxyl, and long-chain alkyl groups of Embelin bind to the peptide backbone and various residues, resulting in the inhibition of probing p300/CBP associated factor (PCAF) lysine acetyltransferase (Modak et al., 2013[47]). One hydroxyl group was alkylated with alkyl, allyl and benzyl derivatives and showed anti-cancer activity against HBL-100 cell lines (Srinivas, 2010[70]). Similarly, the 5-O-methyl-Embelin and 5-O-ethyl-Embelin benzoquinone derivatives exhibited better anti-cancer activity against the different cancer cell lines than human kidney cell lines (Xu et al., 2005[78]). 5-(-chloro-4-trifluoromethoxy-phenylamine derivative of Embelin was reported for lower cell viability against the A375 (melanoma) cell lines (B et al., 2022[4]). Modification of the long chain alkyl group on Embelin with mono and biphenyl alkyl substituents is resulting in specific inhibition of XIAP (X-linked inhibitor of apoptosis protein), a key molecular target for new anticancer agents (Chen et al., 2006[7]). The structural modifications with different substituents on the Embelin revealed the importance of Embelin derivatives (Figure 3(Fig. 3)) for anti-cancer drug discovery (Basha et al., 2022[5]). 

## Bioavailability and Pharmacokinetics of Embelin

Phytotherapy always acts as an alternative or a component of complementary therapy against various chronic disorders. This fact leads the scientific community to study the pharmacokinetics of the phytomolecules to know their bioavailability. Li et al. (2019[42]) studied the pharmacokinetics and oral bioavailability of Embelin in male Sprague Dawley rats (SD Rats) and reported that the oral bioavailability of Embelin is low (30.2 ± 11.9 %) due to its lesser aqueous solubility (Li et al., 2019[42]). In another study, potassium embelate (20 mg/kg) was administered to rats by intravenous and oral routes, and the pharmacokinetics data showed that it followed a 'biexponential kinetic sequence' while given intravenously. In another study, a salt of Embelin, 'Potassium embelate' was administered to the rats to compare the bioavailability of Embelin and Potassium embelate. Interestingly, the absorption of the Potassium embelate was quick and thorough while administered orally (oral bioavailability ~ 97 %), with peak plasma level concentration at 9 g/mL in 0.28 hours. Analyzing the distribution data, it was found that potassium embelate was found accumulated in the brain (after both intravenous and oral administration) (Zutshi et al., 1990[81]). Srinivas et al. (2011[71]) reported that oral administration of 50 mg/kg of Embelin to rats showed a peak plasma concentration of 130.39 ± 6.51 μg/mL after 4.285 hours (Srinivas et al., 2011[71]). Embelin, given orally to athymic nude mice at a dose of 75 mg/kg exhibited the maximal plasma concentration of 3.55 0.13 g/mL after 1 hour of administration. However, a quick decline of the plasma concentration to 0.26 ± 0.06 g/mL at 3 hours was also observed for the same (Edderkaoui et al., 2013[17]). Embelin administered orally (75 mg/kg) to rats exhibited the highest accumulation in kidneys, testes and intestines. Significant levels of Embelin were seen in other organs like heart, spleen and brain. Even after 15 days of Embelin administration, the levels didn't decline, indicating the slow elimination of Embelin from the body (Gupta and Sanyal, 1991[24]).

## Anticancer Mechanisms Exhibited by Embelin: Evidence from Preclinical Studies

Embelin, a natural compound, has shown promising anti-cancer effects against various tumors, including breast, pancreatic, prostate, colorectal, bladder, and liver cancer. Through exploring its distinguished anti-cancer mechanisms, it has been revealed that Embelin induces apoptosis, cell cycle arrest, and autophagy in various cancer cells and animal models. Significantly, Embelin also modulates critical signaling pathways involved in cancer pathophysiology, such as NF-κB, PI3K/Akt, and interleukin-6/STAT3 signaling pathways. These findings highlight the potential of Embelin as a valuable therapeutic agent with multi-faceted anti-cancer activities, targeting critical pathways involved in cancer progression. The preclinical evidence on the anticancer mechanisms of Embelin against various cancers are summarized in Table 1(Tab. 1) (References in Table 1: Dai et al., 2011[8], 2014[9]; Danquah et al., 2009[12], 2012[11]; Dhanjal et al., 2014[15]; Edderkaoui et al., 2013[17]; Fu et al., 2015[21]; Huang et al., 2014[29]; Hussain et al., 2017[30]; Peng et al., 2014[55]) and Table 2(Tab. 2) (References in Table 2: Dai et al., 2009[10], 2011[8], 2014[9]; Danquah et al., 2009[12], 2012[11]; Edderkaoui, et al., 2013[17]; Huang et al., 2014[29]; Hussain et al., 2017[30]; Peng et al., 2014[55]; Sreepriya and Bali, 2005[69]). 

### Apoptosis induction

**Figure 4:**
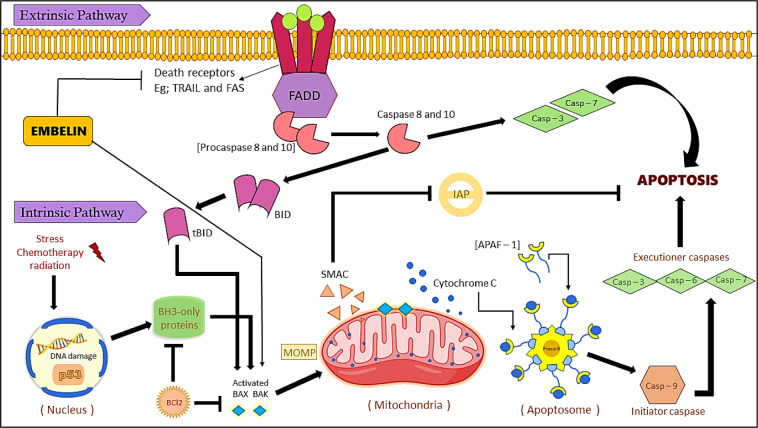
Embelin induces apoptosis through extrinsic and intrinsic pathways in cancer cells. The extrinsic pathway is activated by death receptor ligands (e.g.: TRAIL & FAS). As a result, caspases 8 and 10 execute the downstream pathways, causing apoptosis. The intrinsic apoptosis pathway is activated by BH3 only proteins under stress conditions. This inhibits Bcl-2 proteins, leading to the activation of Bax and Bak. As a result, cytochrome C and Smac are released from mitochondria, forming apoptosomes with APAF-1. This leads to the activation of Caspase 9 and inducing apoptosis.

Apoptosis, a form of programmed cell death, occurs in both physiological and pathological contexts. It is a vital process involved in various cellular functions and is necessary for maintaining a healthy cell cycle. Apoptosis is triggered by cellular damage and irreparable DNA lesions. In the case of cancer, where there is uncontrolled cell division and proliferation, inducing apoptosis in cancer cells serves as an effective mechanism for chemotherapy. By promoting the programmed death of cancer cells, apoptosis can help curb tumor growth and combat the progression of the disease (Morana et al., 2022[48]). Specific morphological changes and energy-dependent molecular pathways characterize the mechanism of apoptosis. Apoptosis is a regulated form of cell death that occurs in response to various stimuli. It induces a series of distinctive cellular and molecular changes, including cell shrinkage, chromatin condensation, DNA fragmentation, and the formation of apoptotic bodies. These changes are orchestrated by a complex network of molecular signaling pathways involving the activation of caspases, regulating Bcl-2 family proteins, and involving death receptors. Through these mechanisms, apoptosis ensures the controlled and orderly elimination of cells, including cancer cells, in response to different triggers or stimuli (Das et al., 2021[13]). Apoptosis, also known as 'programmed cell death', is mediated through two core pathways - intrinsic and extrinsic pathways. Embelin implements apoptosis through both pathways. (Figure 4(Fig. 4)). Caspases are those endoproteases responsible for inducing apoptosis. These cysteine proteases consist of death effector domains which include TNF (tumor necrosis factor), Fas-L (Fas ligand), and TRAIL (tumor necrosis factor-related apoptosis-inducing ligand). Adaptor molecules such as TRADD and FADD bind to these death domains. Other components involved in apoptosis include the amyloid-B peptide, the Bcl-2 family of proteins and the p53 gene (Pfeffer and Singh, 2018[56]).

Extrinsic pathway, also known as death receptor-mediated pathway takes place when the extracellular ligands such as TNF (tumor necrosis factor), Fas-L (Fas ligand), and TRAIL (tumor necrosis factor-related apoptosis-inducing ligand) are connected to the extracellular domain of the transmembrane receptors, death receptors (DR), i.e., the type 1 TNF receptor (TNFR1), Fas (also called CD95/Apo-1) and TRAIL receptors (Elmore, 2007[18]). Embelin regulates the extrinsic apoptotic pathway and blocks the expression of the genes for TNF-α (Tumor necrosis factor-α), TNF receptor-1 (Tumor necrosis factor receptor-1), and TRADD (TNFR1-associated death domain protein). In another research study, it was reported that Embelin reduced the levels of the TNF-α converting enzyme levels in human breast cancer cells through inhibition of MMPs (Matrix metalloproteinases), VEGF (Vascular Endothelial Growth Factor) and hnRNP-K (Heterogeneous nuclear ribonucleoprotein K) molecules in breast cancer cells (MCF 7 and MDA-MB-231 cells). Embelin was found to increase TRAIL-mediated apoptosis in A549 non-small-cell lung cancer cells by lowering the levels of survivin, Bcl-2 (B Cell lymphoma-2), and c-FLIP (Cellular FADD-like IL-1β-converting enzyme-inhibitory protein) (Jiang et al., 2013[34]). Glioblastoma cells were treated with Embelin, tumor necrosis factor-related apoptosis-inducing ligand (TRAIL), or a combination of these two. Malignant glioma cells were widely sensitized to TRAIL-mediated apoptosis after receiving subtoxic doses of the Embelin. Combining Embelin and TRAIL therapy increased the activation of the initiator and effector caspases 8/9 and 3/7, respectively. Embelin also reduced c-FLIP, which made malignant glioma cells more susceptible to TRAIL-mediated apoptosis (Siegelin et al., 2009[66]). Hu et al. (2015[26]) investigated the effects of Embelin (at a lower toxic dose) upon TRAIL-induced apoptosis and its potential mechanism within human leukemia cells. The results indicated that Embelin intensifies the DR4 and DR5 expression making the human leukemia cells more susceptible to TRAIL-induced apoptosis (Hu et al., 2015[26]). In breast cancer cell lines (MDA-MB-231, MCF-7 and MDA-MB-453 cells), Embelin reduced cFLIP_L, a_ regulator in TRAIL-mediated apoptosis (Liang et al., 2021[43]). The intrinsic pathway, the mitochondrial-mediated apoptotic pathway, is triggered by various stimuli such as oxidative stress, irradiation, or treatment with cytotoxic drugs (Jan and Chaudhry, 2019[32]). The intrinsic pathway of apoptosis involves the insertion of Bcl-2 family proteins (XIAP, Mcl-1, Bcl-xL and Bcl-2, Smac, Bak, Bid, and Bax ) into the mitochondrial membrane, resulting in the release of cytochrome C from the mitochondrial intermembrane space into the cytosol, leading to the formation of an apoptosome complex consisting of caspase-9, cytochrome C and apoptotic protease activating factor (Apaf1) (Kim, 2005[36]; Jan and Chaudhry, 2019[32]; Ghobrial et al., 2005[22]). According to the literature, Embelin causes apoptosis through the mitochondria-dependent apoptosis pathway (intrinsic pathway) in a wide range of cancer cells. One of the research studies investigated the molecular mechanisms of apoptosis induction by Embelin in human leukemia cells. The results revealed that Embelin stimulated apoptosis by downregulating XIAP, thus activating caspase-dependent mechanisms in human leukemia cells (Hu et al., 2011[27]). Park et al. (2015[54]) reported that Embelin caused apoptosis to PC3 cells in a time-dependent manner, which was associated with decreased Bcl-2, Bcl-xL, and Mcl-1 expression, increased Bax translocation to mitochondria, and a fall in the mitochondrial membrane potential. Moreover, Embelin stimulated the voltage-dependent anion channel 1 (VDAC) to be expressed and VDAC 1 oligomerization, promoting the release of cytochrome C and apoptosis-inducing factor (AIF).

### Cell cycle arrest

**Figure 5:**
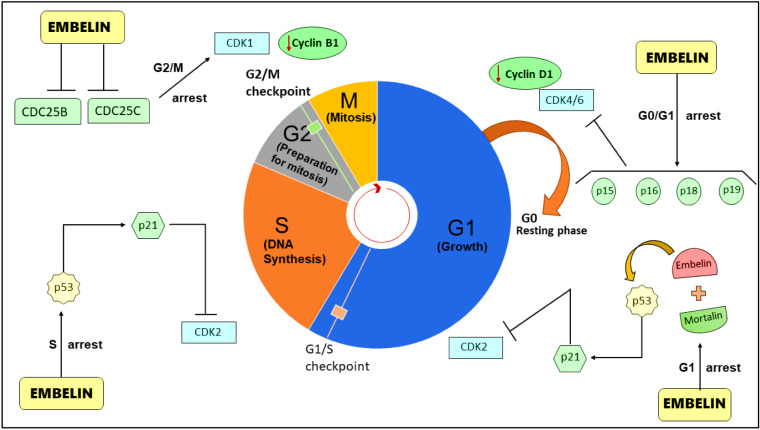
Embelin induces cell cycle arrest at different phases. Embelin is found to decrease the protein levels of CDC25B and CDC25C, causing arrest at the G2 phase. Embelin causes cell cycle arrest at the G1 phase by activating the p53 protein. Embelin causes cell cycle arrest at the G0/G1 phase of the cell cycle by activating the transcription factors p15, p16, p18, and p19, which inhibits CDK4/6 protein levels.

The cell cycle is crucial in controlling cell growth, proliferation, and cell division following DNA damage. It controls the change from quiescence (G0) to cell proliferation and maintains the accuracy of the genetic transcript (Schwartz and Shah, 2005[63]). It is a mechanism that cells use to multiply that is frequently broken down into four stages. There are five stages of the cell cycle- G0 (gap 0), G1 (gap 1), S (DNA synthesis), G2 (gap 2), and M (mitosis). The chief checkpoints between these cell cycle phases include G1/S and G2/M, where the accuracy of DNA synthesis and the integrity of cellular components are observed (Alimbetov et al., 2018[3]). Hindering the cell cycle provides a therapeutic prospect for controlling cancer since cancer cells frequently dysregulate the cell cycle and divide uncontrollably. The majority of anti-cancer medications alter the proliferative cycle of the tumor cells by preventing or disrupting the chief checkpoints of the cell cycle, causing a cell cycle arrest and triggering apoptosis (Senese et al., 2014[64]). Embelin was found to cause a cell cycle arrest in the G0/G1 phase in brain glioma cells (U87 cells). Embelin treatment to U87 cells showed a significant decline in the CDK4, CDK6 and cyclin D1 protein expressions that control the cell cycle (Wang et al., 2013[75]). In human breast cancer cells (MCF 7 and MDA-MB-231), Embelin binds with mortalin (heat shock chaperone present in cancer cells), thus activating the p53 proteins of the tumor cells. The transcriptional activation of p53 causes cell cycle arrest at the G1 phase of tumor cell division (Nigam et al., 2015[50]). In another study, Embelin was found to cause cell cycle arrest at the G2/M phase in osteosarcoma cells (U-2 OS and MG63 cells) (Qian et al., 2018[60]). Embelin-induced cell cycle arrest at S phase and G2/M phases in gastric cancer cells. The molecular analysis in the experiment showed that Embelin reduced the protein expressions of CDK1, cyclinB1, CDK2, CDC25B and CDC25C (Wang et al., 2013[75]). The induction of cell cycle arrest by Embelin is depicted in Figure 5(Fig. 5).

### Autophagy

**Figure 6:**
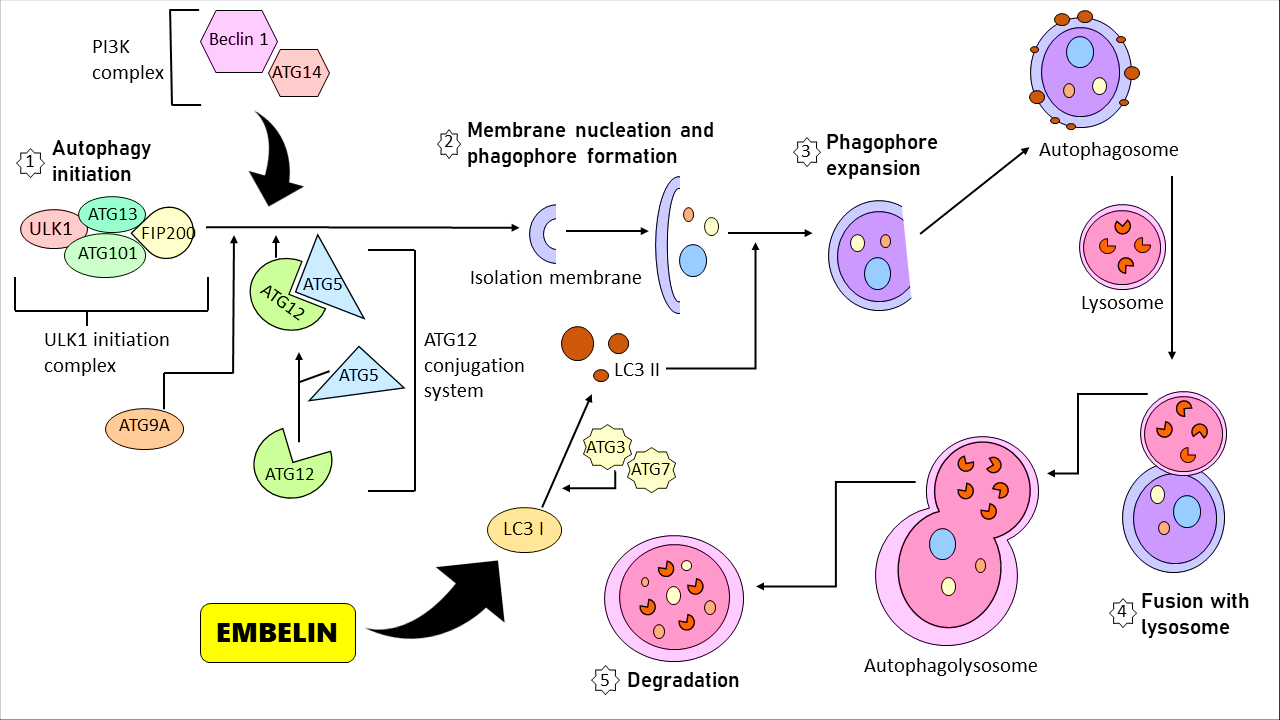
A schematic diagram that explains the autophagic mechanism of cell death and the role of Embelin in autophagy. Several proteins are involved in autophagy, namely ATG. Different ATGs have their potent role in the distinguished steps of autophagy. The steps of autophagy include initiation, phagophore formation and expansion leading to autophagosome formation. This autophagosome fuses with lysosome, leading to degradation. Embelin induces the conversion of LC I to LC II with the help of ATG3 and ATG7 during phagophore expansion.

Autophagy is an intracellular degradative mechanism that occurs under stressful conditions like disruption of an organelle, the presence of aberrant proteins, and nutritional limitation. This intracellular systemic process balances cellular homeostasis and metabolism (Russell et al., 2014[62]). Several proteins regulate the autophagic process. This event starts with the production of 'autophagosomes' (double-membrane vesicles that deliver the degraded components in the cell to the lysosome), which then fuse with lysosomes to recycle the destroyed components (Yun and Lee, 2018[80]). These 'autophagosomes' formation depends on a group of proteins known as 'autophagy-related' proteins or ATG (Yun and Lee, 2018[80]; Onorati et al., 2018[51]). As those internal and external stimulating stimuli cause cells to proliferate, ATG13 binds ULK1 to a pre-autophagosomal structure (PAS), and subsequently nearly all autophagy-related (ATG) proteins assemble hierarchically onto the PAS, which is thought to be an important location for the cytoplasm to vacuole targeting (Cvt) and autophagosome formation (Cao et al., 2021[6]). In these conditions of autophagy induction, the protein kinase complex ULK1/Atg1 (includes ULK1, ATG13, FIP200, and ATG101) plays the role of an 'autophagy initiation complex' (Li et al., 2020[41]). The phosphoinositide-3-kinase complex (PI3K) gets clustered with the PAS taking part in the formation of 'phagophore' or 'isolation membrane' via the interaction and binding of ATG14L with ATG13 at PAS (Yang et al., 2021[79]; Kocaturk et al., 2019[38]). As the initial tiny ATG9A vesicles join PAS forming a phagophore, the 'bowl-shaped' double membrane gradually elongates and wraps around pieces of cytoplasm and organelles (Militello and Colombo, 2011[45]). These mature autophagosomes fuse with lysosomes forming autophagolysosomes (Wijdeven et al., 2016[77]). Autophagy performs dual roles in cancer progression and suppression (Lim and Staudt, 2013[44]; Morel et al., 2017[49]). The role of Embelin in inducing autophagy is represented in Figure 6(Fig. 6). Embelin induced autophagy in human tongue squamous cells (Ca9-22 cells). On Embelin treatment, LC3‐I converted to LC3‐II and the degradation of several autophagy-related proteins was also observed - ATG5‐ATG12 complex, p62/SQSTM1 and Beclin‐1 (Lee et al., 2017[40]) - embelin-induced autophagy in ovarian cancer cell lines, identified through orange staining. The results showed an accumulation of GFP-LC3 proteins and Beclin-1, autophagy biomarkers (Jehan et al., 2012[33]; Poojari, 2014[58]). 

### Other signal transduction pathways

The literature search on the anti-tumor mechanisms of Embelin revealed that apart from the major anticancer mechanisms like apoptosis, cell cycle arrest and autophagy, some of the signal transduction pathways involved in cancer pathophysiology are also regulated by Embelin (Table 3(Tab. 3); References in Table 3: Ahn et al., 2007[1]; Cao et al., 2021[6]; Dai et al., 2014[9]; Das et al., 2021[13]; Elmore 2007[18]; Elwakeel, 2022[19]; Ghobrial et al., 2005[22]; Heo et al., 2011[25]; Hu et al., 2015[26]; Israël, 2010[31]; Jan and Chaudhry, 2019[32]; Jehan et al., 2012[33]; Jiang et al., 2013[34]; Kim, 2005[36]; Kocaturk et al., 2019[38]; Lee et al., 2017[40]; Li et al., 2020[41]; Liang et al., 2021[43]; Militello and Colombo, 2011[45]; Mizukami et al., 2002[46]; Morana et al., 2022[48]; Nigam et al., 2015[50]; Onorati et al., 2018[51]; Park et al., 2015[54]; Pfeffer et al., 2018[56]; Poojari et al., 2014[58]; Prabhu et al., 2017[59]; Qian et al., 2018[60]; Russell et al., 2014[62]; Siegelin et al., 2009[66]; Soubannier and Stifani, 2017[68]; Wang et al., 2013[75]; Wijdeven et al., 2016[77]; Yang et al., 2021[79]; Yun and Lee, 2018[80]). The Nuclear Factor-kB is a dimeric DNA-binding complex consisting of various homo- and heterodimers of Rel family protein harboured by the I*k*B family of repressive proteins that conceals the nuclear localisation signal and keeps them sequestered in an inert state (Soubannier and Stifani, 2017[68]). This transcription factor plays a crucial role in various physiologic processes, mainly inflammation, the body's defense mechanisms, cell growth and viability (Park et al., 2015[54]). Different stimuli such as cytokines (TNF-α, IL-1β), growth factors [epidermal growth factor], microbiological products [lipopolysaccharide, dsRNA], various ionizing and non-ionizing radiation, oxygen free radical, genetic mutations and intracellular oncogenic stress activates NF-κB signaling. The IκB molecules become phosphorylated in response to various inflammatory mediators or due to any foreign particles, which in turn break down the NF-κBs and the free NF-κBs thus formed enter the nucleus and activate the transcription of a variety of genes participating in the immune and inflammatory response, cell adhesion, growth control, and protection against apoptosis (Israël, 2010[31]). The receptor activator of NF-κB and its ligand RANKL stimulates NF-κB through IκB kinase activation (Mizukami et al., 2002[46]). A study conducted to assess the therapeutic efficacy of Embelin, a potential inhibitor of the RANKL cell signaling pathway against invasive glioma showed that Embelin induced apoptosis by inhibiting the NF-kB and thereby suppressed the progression of glioma cell lines in a dose and time-dependent manner without affecting the normal cells (Park et al., 2015[54]). Embelin adversely impacted the protein expression involved in cell survival, proliferation, invasion, and tumor metastasis due to the increased rate of apoptosis (Ahn et al., 2007[1]). Akt, belonging to the family of phosphatidylinositol-3-OH-kinase-regulated serine/threonine kinase, contributes to cell survival and represses apoptosis in various cell types brought about by various stimuli, cell cycle dissentients, and cell detachment. Embelin was found to inhibit the binding of XIAP (an anti-apoptotic protein) to the initiation caspase, thereby producing cytotoxic effects by reducing the activity of various signaling pathways, mainly PI3Kinase/AKT pathway in different cancer cells (Prabhu et al., 2017[59]). Embelin was found to inhibit the extensive proliferation of osteosarcoma and induced apoptosis by PI3K/Akt pathway, and suppress XIAP, leading to the termination of caspase activation (Qian et al., 2018[60]). In an attempt to study the effect of Embelin on various human cancer cell lines, a decrease in cell viability was observed in the Embelin-treated cells, particularly the prostate cancer cells, in a dose-dependent manner without affecting the normal cells by inhibiting the Akt signaling pathway and thereby leading to apoptosis (Park et al., 2015[54]). Another important oncogenic inhibitor is the tumor suppressor gene, p53. The stress chaperone, mortalin (inactivation of tumor suppressor p53 and PI3K/ AKT) treated with Embelin, activates p53 by causing nuclear translocation followed by transcriptional activation and causes suppression of cancer cell growth (Nigam et al., 2015[50]; Elwakeel, 2022[19]). The protumorigenic effect of the interleukin-6**/**STAT3 signaling pathway significantly impacts the pathophysiology of inflammatory bowel disease and colorectal cancer (Dai et al., 2014[9]). Several research studies carried out suggested that the prolonged activation of STAT3 working along with NF-κB promotes tumour development. Hence, its inhibition can lead to regression in tumor growth and proliferation. The inhibition of the actuation of JAK2 and c-Src by Embelin suppresses the activity of STAT3. The protein expression of STAT3 is down-regulated by Embelin, henceforth activating caspase-3 and inducing apoptosis, inhibiting the proliferation of cancer cells (Heo et al., 2011[25]).

## Toxicity, Side Effects and Safety of Embelin

In terms of toxicity, 3 g/kg of Embelin is harmless. No subacute oral toxicity was identified even though the rats were given 10 mg/kg Embelin for ten weeks (Ko et al., 2018[37]). A dose of 50 and 100 mg/kg of Embelin did not indicate a notable toxic effect or a change in body weight, thus marking its safety outline (Kundap et al., 2017[39]). Female rats that were given Embelin at 120 mg/kg per day for six weeks generated adrenal hypertrophy, kidney damage and the decomposition of hepatocytes (Li et al., 2019[42]). No change was observed in the protein and glycogen levels in the kidney, spleen, and liver. However, a significant increase was observed in the levels of these constituents in the adrenal gland. Additionally, the kidney and adrenal glands observed elevated activity of both acid and alkaline phosphatase enzymes. For 14 weeks, Wistar rats were given 50 mg/kg/day of Embelin, but it failed to create a significant fall in blood counts; instead, the hematopoietic cells were afflicted (Kundap et al., 2017[39]). In the case of *in vitro *cytotoxic studies, after 72 hours of incubation, the 20 μg/mL of Embelin indicated no effect on human fibroblasts. Sarcoma cells were more sensitive to it than murine melanoma cells, but normal cells were unaffected (Podolak et al., 2005[57]). A review of the toxicity studies imparted that Embelin was safe and non-toxic at therapeutic doses. In contrast, some toxicity is expressed at higher doses (above LD_50, _i.e. 44 mg/kg) (Kundap et al., 2017[39]).

## Limitations

Despite the advancements in understanding the role of natural bioactive compounds as potential anticancer agents, there are several multidimensional challenges that hinder their widespread use as primary therapies for cancer. These compounds are often utilized as adjuvants or combined with conventional chemotherapy rather than standalone treatments. Some of the key challenges include:

*Limited clinical evidence*: While preclinical studies have shown promising anticancer effects of natural bioactive compounds, there is often a lack of comprehensive clinical evidence to support their efficacy and safety in human cancer patients. Rigorous clinical trials are necessary to establish their effectiveness and determine optimal dosages.

*Bioavailability and pharmacokinetics*: Natural bioactive compounds may have poor bioavailability, limited solubility, and inadequate tissue penetration, affecting their therapeutic efficacy. Optimizing their formulation, delivery systems, and pharmacokinetic profiles is essential for enhancing their bioavailability and targeting cancer cells specifically. Indeed, the clinical use of Embelin has been limited by its poor aqueous solubility. However, when administered in the form of its salt, Potassium embelate, it demonstrates improved absorption. The anticancer activity of Embelin is attributed to the presence of its benzoquinone core in its chemical structure. To overcome the solubility limitations, researchers have attempted to modify the structure of Embelin to enhance its aqueous solubility. Several structural modifications have been investigated, but none of these modifications have proven to be effective enough to make Embelin clinically useful. The challenge of improving the aqueous solubility of Embelin remains an ongoing area of research to fully harness its potential as an anticancer agent for clinical applications.

*Standardization and quality control*: Natural bioactive compounds are derived from diverse sources, and their chemical composition can vary significantly. Standardization of these compounds is challenging, making it difficult to ensure consistent quality, potency, and efficacy. Robust quality control measures are needed to address these issues.

*Drug Resistance and Side Effects:* Similar to conventional chemotherapy, natural bioactive compounds may face the challenges of drug resistance and adverse side effects. Developing strategies to overcome drug resistance and minimize toxicity is crucial for their successful use in cancer treatment. 

*Regulatory and commercialization challenges*: Regulatory hurdles and the lack of financial incentives for developing natural bioactive compounds as standalone therapies pose significant challenges. The complex regulatory landscape and limited market exclusivity impede their development and commercialization. Addressing these multidimensional problems requires collaborative efforts among researchers, clinicians, regulatory agencies, and pharmaceutical companies. Continued research, clinical trials, and innovative approaches are needed to unlock the full potential of natural bioactive compounds as effective and safe anticancer therapies.

## Conclusions and Future Perspectives

Embelin, a natural bioactive compound, has garnered considerable attention for its potential anticancer effects. In recent years, there has been a renewed interest in revisiting and further exploring the anticancer properties of Embelin, to facilitate its clinical translation. This updated review revisits Embelin's anticancer effects and aims to bridge the gap between preclinical research and clinical applications. Numerous studies have highlighted the multifaceted mechanisms by which Embelin exerts its anticancer effects. It has been shown to possess anti-proliferative, pro-apoptotic, anti-metastatic, anti-angiogenic, and anti-inflammatory properties. Embelin targets various signaling pathways in cancer progression, including NF-κB, PI3K/ Akt, and STAT3. These pathways play crucial roles in tumor growth, survival, angiogenesis, and metastasis. Several challenges need to be addressed to facilitate the clinical translation of Embelin. These include enhancing its bioavailability, optimizing its formulation and delivery systems, determining appropriate dosages, and ensuring its safety and efficacy in clinical settings. Furthermore, conducting well-designed clinical trials is crucial to provide robust evidence of Embelin's anticancer effects and evaluate its potential as a standalone therapy or in combination with conventional treatments. Additionally, efforts should focus on identifying biomarkers and patient selection criteria to help predict and monitor the response to Embelin treatment. This personalized approach can improve the effectiveness and safety of Embelin-based therapies. Toxicity studies on Embelin suggested that the administration of 100 mg/kg Embelin showed no remarkable harmful effects. Still, the results varied greatly depending on the animal model under consideration, yet Embelin has proved to be a potent phytomedicine against various cancer cell lines at the preclinical stage. In conclusion, revisiting the anticancer effects of Embelin holds promise for its clinical translation. Further research, including well-designed clinical trials, is needed to establish the optimal use of Embelin as a standalone or adjunct therapy for various types of cancer. By addressing the challenges associated with its clinical translation, Embelin has the potential to emerge as a valuable anticancer agent that can benefit cancer patients.

## Notes

Adithya Jayaprakash Kamath, Alda Sara Chandy, Aina Ann Joseph and Jaggiah N. Gorantla contributed equally as first author.

Lekshmi R. Nath, Javad Sharifi-Rad (Facultad de Medicina, Universidad del Azuay, Cuenca, Ecuador; E-mail: javad.sharifirad@gmail.com) and Daniela Calina (Department of Clinical Pharmacy, University of Medicine and Pharmacy of Craiova, 200349 Craiova, Romania; E-mail: calinadaniela@gmail.com) contributed equally as corresponding author.

## Declaration

### Competing interests

The authors wish to confirm that there are no known conflicts of interest associated with this publication, and there has been no significant financial support for this work that could have influenced its outcome.

### Funding

Not applicable.

### Authors' contributions

AJK, ASC, AAJ, JNG, ADD, LRN, JS-R and DC made a significant contribution to the work reported, whether that is in the conception, study design, execution, acquisition of data, analysis, and interpretation, or in all these areas that is, revising or critically reviewing the article, giving final approval of the version to be published; agreeing on the journal to which the article has been submitted; and confirming to be accountable for all aspects of the work. All authors have read and agreed to the published version of the manuscript.

## Figures and Tables

**Table 1:**
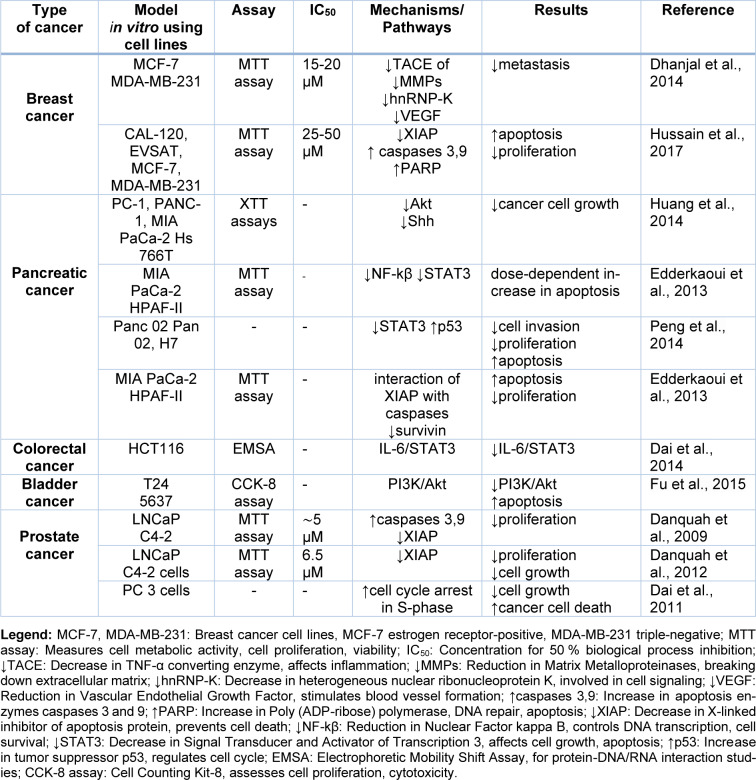
*In vitro* studies of Embelin against cancer

**Table 2:**
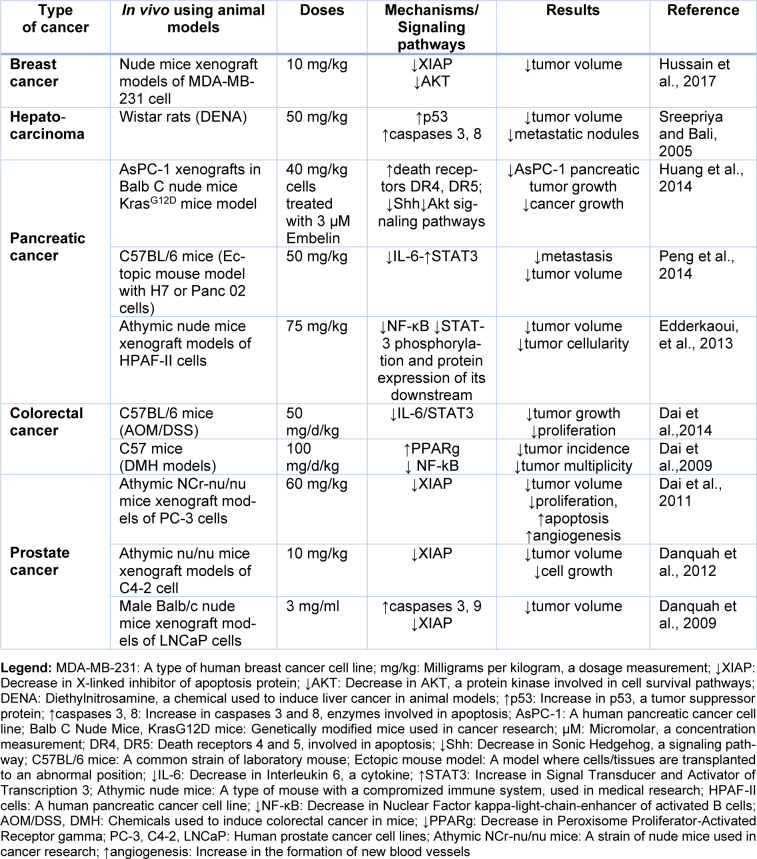
*In vivo* studies of Embelin against cancer

**Table 3:**
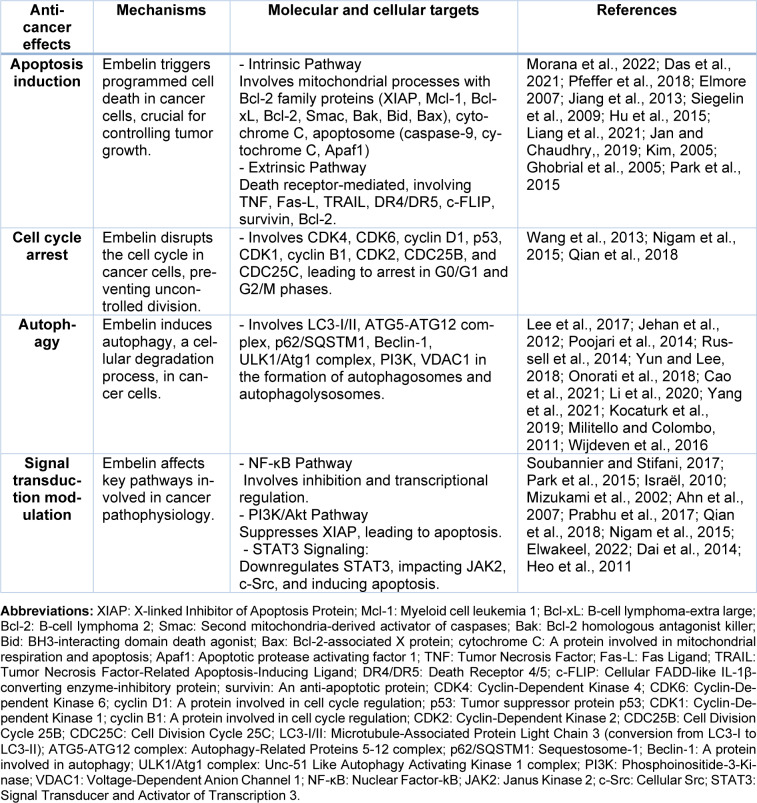
Summarizing the key mechanisms, including apoptosis induction, cell cycle arrest, autophagy, and the modulation of crucial signal transduction pathways, alongside their molecular components of Embelin

**Figure 1:**
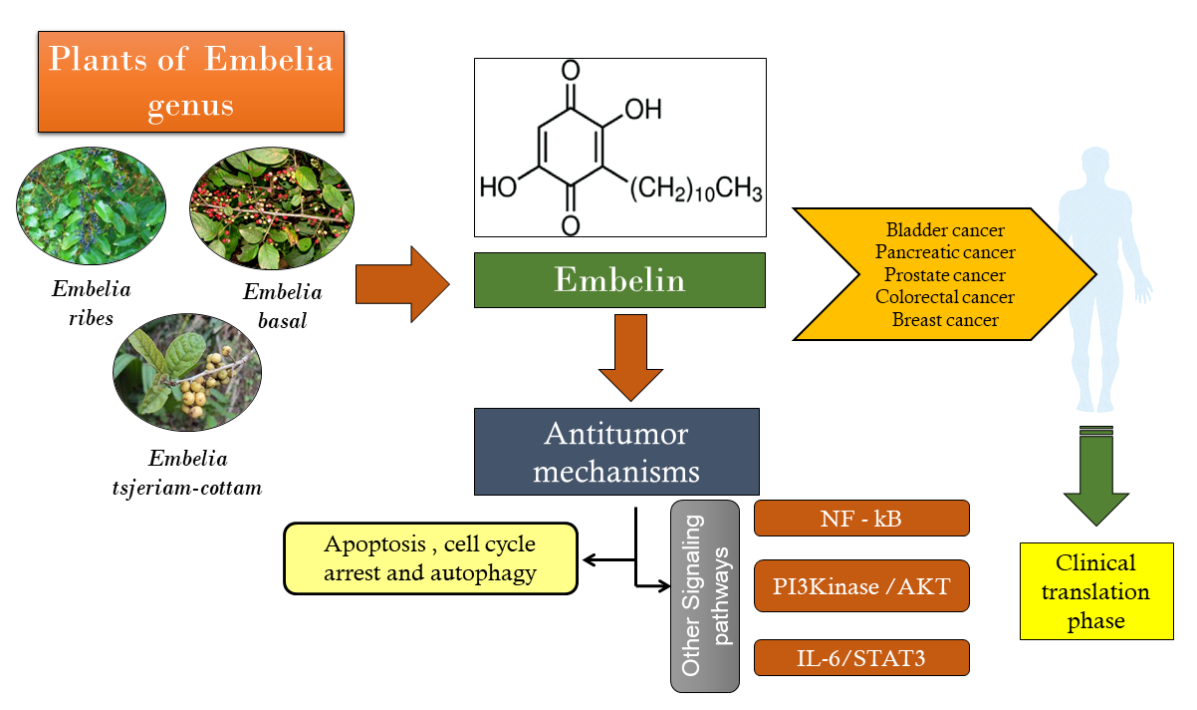
Graphical abstract
